# Long-Term Prediction of Mesoscale Sea Surface Temperature and Latent Heat Flux Coupling Using the iTransformer Model

**DOI:** 10.3390/s25030985

**Published:** 2025-02-06

**Authors:** Xuwei Hu, Yuan Feng, Jiahao Liu, Yuanxiang Xu, Shengyu Song

**Affiliations:** Faculty of Information Science and Engineering, Ocean University of China, Qingdao 266100, China; huxuwei@stu.ouc.edu.cn (X.H.); liujiahao6266@stu.ouc.edu.cn (J.L.); xuyuanxiang@stu.ouc.edu.cn (Y.X.); 21210213114@stu.ouc.edu.cn (S.S.)

**Keywords:** mesoscale SSTa–LHFa coupling, iTransformer model, western boundary currents, climate variability prediction, sea surface temperature, latent heat flux, linear trend

## Abstract

Mesoscale air–sea interaction, which is active in Western Boundary Currents (WBCs), has a non-negligible effect on mid-latitude climate variability. The analysis and prediction of the mesoscale air–sea interaction rely on high-resolution observation datasets and mesoscale-resolving climate models, which often require long processing times to estimate future changes and have several limitations. Therefore, in this study, we used a newly developed iTransformer model, which integrates mesoscale sea surface temperature anomaly (SSTa) and latent heat flux anomaly (LHFa) coupling coefficient data to predict future changes in SSTa–LHFa coupling. First, we individually trained the model using data corresponding to 1–15 past winters from ERA5 dataset. Thereafter, we used the trained model to predict SSTa–LHFa coupling coefficient for the next 10 winters. Compared with the predictions using only the coupling coefficient, the prediction yields 3.0% relative improvements when SST data were incorporated. The iTransformer model also showed the ability to reproduce the linear trend and mean value of mesoscale SSTa–LHFa coupling coefficients. Furthermore, we chose the optimal input length for each WBC and used the model to predict changes in mesoscale SSTa–LHFa coupling in the future. The results thus obtained were comparable to those obtained using mesoscale-resolving climate models, indicating that the iTransformer model showed satisfactory prediction performance. Therefore, it provides a novel pathway for exploring mesoscale air–sea interaction variations and predicting future climate change.

## 1. Introduction

Ever since the early years of the 21st century, satellite altimeters have provided critical observational datasets with resolutions higher than those used in existing climate models. Thus, they offer the possibility to improve the representation of oceanic mesoscale variability [1,2]. It has also been demonstrated that large-scale air–sea interaction is influenced by atmospheric forcings [3,4] and that the mesoscale effects of ocean forcings on the atmosphere are positively associated with surface wind speed and sea surface temperature (SST) [2,5,6,7,8,9]. Additionally, mesoscale oceanic eddies have significant thermodynamic effects on marine boundary layers, as well as on turbulent heat flux anomalies (THFa), precipitation, and cloud liquid water [10,11,12,13]. These thermodynamic effects have been verified using reanalysis datasets and have also been employed in the development of high-resolution climate models [2,14,15], which have provided insights that have increasingly drawn attention to mesoscale air–sea interaction in mid-latitude western boundary currents (WBCs) and enhanced understanding regarding mid-latitude climate variability [16,17,18,19,20].

The most prominent mesoscale air–sea interaction is the positive correlation between mesoscale SST anomalies and THF anomalies (SSTa–THFa), hereafter referred to as thermal coupling. Reportedly, thermal coupling is a critical trigger and modulator in mid-latitude air–sea interaction systems. On the one hand, it supplies heat to the atmosphere, resulting in local changes in atmospheric surface wind, precipitation, cloud characteristics, and heat fluxes [2,4,5,6,7,8,9]. From a large-scale perspective, mesoscale oceanic processes significantly modify synoptic storm tracks, atmospheric rivers, and large-scale atmospheric circulations, thereby influencing sub-seasonal to seasonal weather prediction [21,22,23,24]. On the other hand, thermal coupling dampens SST and eddy potential energy, which plays a key role in maintaining the oceanic fronts and can drive changes in large-scale oceanic circulations and alter water formation. Therefore, thermal coupling plays a critical role in influencing long-term climate variability [17,25,26,27,28,29]. Additionally, based on these previously reported findings, the coupling strength of mesoscale SSTa–THFa interaction represents the intensity of mesoscale air–sea interaction [21]. It can also serve as a crucial coupler and modulator in the air–sea interaction system. A deeper understanding in this regard may lead to the improved predictions of future changes in ocean and atmosphere climates.

Putrasahan et al. (2013) investigated the impact of SST distribution on THF by analyzing the linear relationship between LHF and SST and represented the regression coefficient of this relationship as the coupling coefficient [20]. Ma et al. (2016) identified significant seasonal variations in the responses of surface wind speed and heat fluxes to oceanic eddies in the Kuroshio Extension (KE) region, as revealed through their analysis of the SSTa–LHFa coupling coefficient [10]. Even though several attempts have been made to confirm the important role of mesoscale thermal air–sea coupling in climate variability, understanding of future mesoscale thermal coupling remains limited. Moreover, given that prediction analysis relies heavily on long-term high-resolution mesoscale-resolving climate models, which are relatively scarce and require long processing times, we directly predicted changes in mesoscale thermal SSTa–LHFa coupling using the iTransformer model based on ERA5 reanalysis datasets for four major WBCs (KE, Gulf Stream (GS), Agulhas Return Current (ARC), and Brazil–Malvinas Current (BMC)). Given that the coupling strength peaks during winter for both hemispheres, in this study, we aimed to investigate possible variations in the strength of mesoscale SSTa–THFa coupling and train the coupling coefficient time series in WBCs over the past 39 winters using the iTransformer model. Furthermore, we compared the linear trend between iTransformer model-based predictions and those based on the original coupling coefficient time series and reproduced the coupling coefficient time series for the latest 10 winters. Additionally, we compared the projected coupling coefficients obtained using High-Resolution Model Intercomparison Project (HighResMIP) simulations under global warming with those obtained using the iTransformer model. The findings of this study may provide valuable insights on future changes in mesoscale air–sea interaction.

For long-term time series forecasting (LTSF) tasks, the primary methods previously employed were convolutional neural networks (CNNs) and recurrent neural networks (RNNs). CNN-based models extract temporal information by moving convolutional kernels across a time series, whereas RNN-based networks handle the context of a time series by introducing gating mechanisms into the recurrent structure. Methods based on CNNs and RNNs have been widely applied in the field of air–sea interactions. Yuan et al. (2019) applied a convolutional LSTM (ConvLSTM) network combined with ensemble empirical mode decomposition to daily forecasts of the North Atlantic Oscillation (NAO) [30]. Ham et al. (2021) proposed an all-season convolutional neural network (A_CNN) model to predict the El Niño–Southern Oscillation (ENSO) index for boreal spring, which is the most challenging season to forecast [31]. Li et al. (2022) used ConvLSTM to predict SST for the next seven months and calculate the Indian Ocean Dipole (IOD) index, with results showing a correlation of 82% [32]. However, CNNs tend to focus more on extracting local feature information, which limits their ability to capture the global features of time series data. Although RNNs perform well in short-term prediction tasks, as the prediction time span increases, they are prone to gradient explosions or vanishing, and their inference speed decreases rapidly. The Transformer model [33], a deep neural network architecture based on the self-attention mechanism, was initially used for natural language processing (NLP) and has since been extensively applied in fields such as Computer Vision (CV) and audio processing. The attention mechanism endows the Transformer model with superior semantic feature extraction capabilities. As a result, it demonstrates excellent performance with respect to long-term dependency relationships and has enormous potential for application in LTSF tasks. Additionally, experimental results based on multiple real-world datasets have shown that the Informer model [34] based on probabilistic attention, whether in univariate or multivariate LTSF tasks, outperforms both RNN-based long and short-term memory (LSTM) and CNN-based Long- and Short-term Time-series network (LSTNet) models, and has a significantly reduced root-mean-square error (RMSE). In recent years, various transformer-based solutions, such as LogTrans [35], Autoformer [36], and frequency-enhanced decomposed transformers (FEDformer) [37], have emerged for LTSF. However, the self-attention mechanism itself is permutation invariant, implying that it pays more attention to the semantic features of the time series than it does to the order of tokens in the series. Moreover, real-world time-series data are often non-continuous, possibly owing to missing values or the fact that they only focus on data corresponding to a specific time period based on specific needs. This invariance may cause the Transformer to lose some temporal information during the processing of time-series data, hence its application potential is limited. Recent studies [38,39] have shown that linear mapping with reversible instance normalization (RevIN) [40] enables the extraction of periodic and trend information from long-term time series data, and the larger the look-back window, the more temporal information is obtained. Liu et al. (2023) re-examined the functioning of the attention mechanism in the Transformer model and proposed that focusing the attention mechanism on capturing the correlation of multivariate series and using linear mapping to model the time-series information (iTransformer) can yield better performance results [41]. Additionally, by using the modified model to analyze multiple real-world datasets, they obtained state-of-the-art performance. Thus, their work indicated that it is possible to significantly expand the applicability of transformers in the LTSF field. Models like RNNs and CNNs are often less efficient for long sequences due to their difficulty in capturing long-range dependencies and their computational bottlenecks. In contrast, the iTransformer benefits from a parallelizable architecture that accelerates computation, particularly when processing large datasets. Its architecture is also scalable, allowing it to handle larger datasets without a significant loss in performance. Moreover, the iTransformer can seamlessly manage multivariate time series data, a capability that CNNs and RNNs lack. Therefore, in this study, we used the iTransformer model to predict the mesoscale SSTa–LHFa coupling coefficient.

## 2. Materials and Methods

### 2.1. Dataset

**Figure 1:**
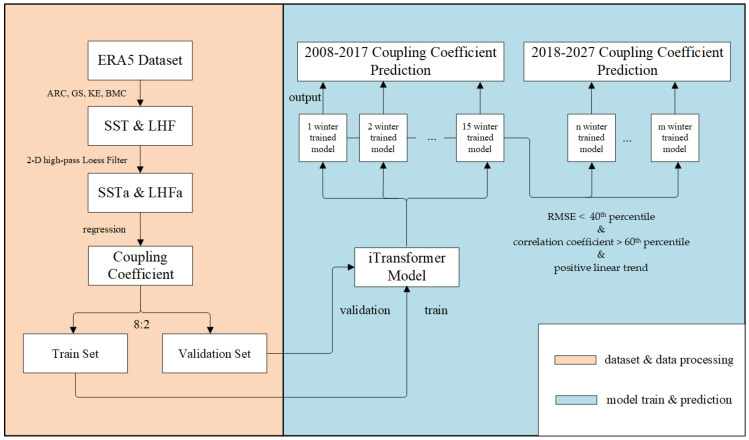
Research framework.

Figure 1 shows the research framework of this paper. In this study, we utilized the recently released fifth-generation, 6-hourly, 0.25° resolution, 2-D surface ERA5 global atmospheric reanalysis dataset for the period 1979–2017. The dataset, titled “ERA5 post-processed daily statistics on single levels from 1940 to present”, was provided by the European Centre for Medium-Range Weather Forecasts (ECMWF) [42]. ERA5 represents a significant improvement in the characterization, intercalibration, and processing of conventional and satellite measurements. These enhancements have progressively refined the quality of historical observations, particularly in terms of coverage and accuracy. The dataset provides high-resolution atmospheric data, including sea surface temperature (SST) measured in degrees Celsius (°C) and latent heat flux (LHF) measured in watts per square meter (W/m^2^), both of which are crucial for analyzing mesoscale air–sea interaction.

### 2.2. Data Processing

The original dataset has a temporal resolution of 6 h; however, for our analysis, we processed the data to derive daily averages by calculating the mean values of SST and LHF. We applied a 2-D spatial high-pass Loess Filter with a cutoff wavelength of 15° longitude and 5° latitude for data filtering to remove low-frequency background signals and retain mesoscale variability. This filter is based on a locally weighted quadratic regression and yields a half-power cutoff at the corresponding value in its half-sized window. To obtain mesoscale anomalies, we eliminated the smooth fields from the original SST and LHF data:

$$\mathrm{SSTa}=\;\mathrm{SST}\;-\;{\bar{\mathrm{SST}}}_{\mathrm{filter}}$$

(1) $$\mathrm{LHF}a=\;\mathrm{LHF}\;-\;{\bar{\mathrm{LHF}}}_{\mathrm{filter}}$$

**Figure 2:**
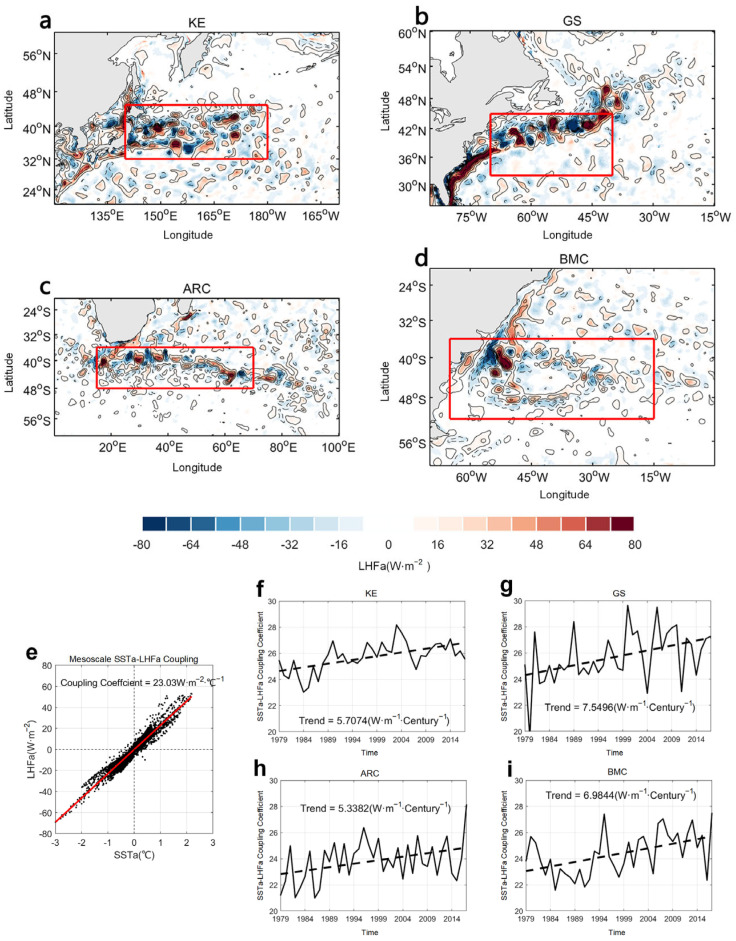
Spatial characteristic of high-pass filtered SSTa (contour) and LHFa (color) at four WBCs (red box) in ERA5 (**a**–**d**). Scatterplots with the slope of mesoscale LHFa against mesoscale SSTa in KE (e.g., (**e**)). Time series of mesoscale SSTa–LHFa coupling coefficient (solid line) and linear trend (dashed line) using wintertime average datasets in KE (**f**), GS (**g**), ARC (**h**) and BMC (**i**). All calculations were carried out using hemispheric wintertime average data and the regression coefficient is significant at 5% level.

The spatial characteristics of the mesoscale SSTa and LHFa in ERA5 during the hemispheric wintertime of 1984 (December–February (DJF) in the Northern Hemisphere and June–August (JJA) in the Southern Hemisphere) are shown in Figure 2a–d. This figure also illustrates the in-phase correlation between the mesoscale SSTa and LHFa in the four WBCs. We then regressed LHFa onto SSTa to obtain the regression coefficients. The regression coefficients depict changes in LHF in response to a 1 °C change in SST at oceanic mesoscales. Furthermore, the regression coefficients represent the damping of SST by LHF and provide an estimate of the LHF ocean heat gain (or loss) per degree increase in SST [43,44]. Thus, the linear regression coefficients of the mesoscale SSTa–LHFa interaction represent the strength of mesoscale air–sea interaction. Thus, we performed regression analyses for the KE (140–180° E, 32–45° N), GS (70–40° W, 32–45° N), ARC (10–70° E, 36–48° S), and BMC (55–85° W, 36–52° S). Figure 2e shows the regression relationship between the winter average mesoscale SSTa and LHFa for 1984 in the ARC. Additionally, for better visualization, we constructed a time series of coupling strength indices by calculating the mean winter value for each year in each WBC as shown in Figure 2f–i. This figure also shows a significant increasing trend in the coupling indices for the selected 39 winters (1979–2017, significance at the 5% level). These increasing linear trends observed in the four WBCs implied that, over the past 39 winters, mesoscale air–sea interaction became increasingly active. This greater activity potentially influenced mid-latitude climate variations, as well as their predictability. Thus, in this study, we also used coupling coefficients and linear trends for predictive analysis. Specifically, we performed linear regression analyses to capture linear trends within the 1979–2017 data and, thereafter, performed data training using a time series constructed based on daily coupling coefficients. The use of daily datasets enabled the reproduction of mesoscale characteristics and linear trends as shown in Figure 2f–i similar to those shown in Figure 2a–d.

### 2.3. iTransformer Model

**Figure 3:**
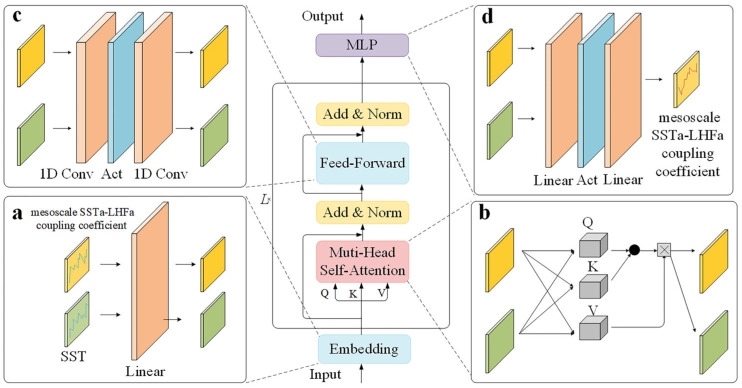
Overview of iTransformer architecture. The model consists of Embedding layer, Encoder Layer and MLP projection Layer: (**a**) The original multivariate time series is embedded to tokens through a Linear layer. (**b**) Embedded tokens are processed through Multi-Head Self-Attention (MHSA) to extract correlations between different variables. (**c**) Feed-Forward network (FFN) layer further captures the local features of processed series. (**d**) Finally, an MLP maps the extracted tokens into the predicted series.

Generally, two strategies are used in the prediction process: The first is iterated multistep (IMS) forecasting, the second is direct multistep (DMS) forecasting. Owing to the inevitable error accumulation associated with IMS forecasting, which becomes more significant as the prediction range increases, we adopted a DMS prediction strategy instead of IMS prediction. Thus, the entire model consists of embedding, encoder, and multilayer perception (MLP) layers. Unlike the encoder–decoder architecture of the vanilla Transformer, the iTransformer is based on an encoder-only structure, as shown in Figure 3. This not only reduces the computational overhead of the transformer but also enables the model to focus more on representing learning and adaptive correlations within the multivariate series. Thus, the entire model is concise, lightweight, and easy to expand.

The embedding layer transforms the input, **X** ∈ ℝ^2×*T*^, which consists of mesoscale SSTa–LHFa coupling coefficient and SST, into a feature vector, **H**_0_ ∈ ℝ^2×*N*^, via a learnable linear function. *T* represents the look-back window size and *N* represents the internally processed dimension using the encoder. Notably, there is no need to add extra positional or temporal features, as is the case with the vanilla Transformer, because this information can be naturally modeled by the subsequent MLP layer.

(2) $$H_{0}=\mathrm{Embedding}\left(X\right)$$

**H**_0_, obtained via embedding, was passed through the *L* layers of the encoder to obtain the hidden feature of the model as shown in Equation (3).

(3) $$H_{l+1}=\mathrm{Encoder}\left(H_{l}\right),l=0,\ldots ,L-\;1$$

Each encoder layer included multi-head self-attention (MHSA) and feedforward network (FFN) sublayers, with residual connection (Add) and layer normalization (LN) performed after passage through each sublayer. Furthermore, the Add and LN steps were performed to ensure the stability of data distribution and accelerate the convergence speed of the model.

$$H_{l}^{\prime }=\mathrm{LN}\left(\mathrm{MHSA}\left(H_{l}\right)+H_{l}\right)$$

(4) $$H_{l+1}=\mathrm{LN}\left(\mathrm{FFN}\left(H_{l}^{\prime }\right)+H_{l}^{\prime }\right)$$

MHSA functioned as the core of the iTransformer model as it enabled the model to focus on the time series information of variables of different dimensions by mapping **H***_l_* to h different semantic spaces (i.e., **Q***_i_*, **K***_i_*, **V***_i_* ∈ℝ^2×*d*^, 1 ≤ *i* ≤ *h* and *d* = *N*/*h*). The FFN sub-layer consisted of two 1D convolutional blocks (1D Conv) and a ReLU activation function. The 1D Conv, with a kernel size of 1, was used to extract the correlations and local features within the multivariate time series. The output **A***_i_* of the multihead was then concatenated and returned to *N* dimensions. Next, MHSA was calculated as shown in Equation (5).

$$A_{i}=\mathrm{Softmax}\left(Q_{i}{K_{i}}^{Τ}/\sqrt{d}\right)V_{i}$$

(5) $$\mathrm{MHSA}\left(H_{l}\right)=\mathrm{Concat}\left(A_{1},\ldots ,A_{h}\right)$$

MLP employs two functions, the linear mapping and Gaussian error linear unit (GELU) activation functions. The linear mapping function plays a crucial role in long-term time prediction and exhibits robustness against an increase in input length for multivariate time series. This allowed the model to better learn cyclical patterns and trend information within the data.

(6) $$Y=\mathrm{MLP}(H_{l})$$

where **Y** ∈ ℝ^1×*S*^ and *S* represents the forecasting length of the mesoscale SSTa–LHFa coupling coefficient.

## 3. Results

### 3.1. Experimental Parameter Settings

During the prediction of coupling coefficients for time-series data, the size of the look-back window (i.e., the input length) had varying effects on the output of the model. A smaller look-back window allowed for a larger partition of the training data but limited the historical information available. Conversely, a larger input length provided more extensive historical information but reduced the volume of the partitioned training data. The size of the look-back window also had a significant effect on the prediction accuracy of the model. Given the substantial impact of the look-back window size on prediction accuracy, we balanced the trade-off between training data size and input length by using daily coupling coefficients from the past 1–15 winters as input to predict daily coupling coefficients for the next 10 winters. This approach generated 15 output time series for each WBC.

For model optimization, we employed AdamW with a learning rate of 0.001 and a batch size of 32, along with an early stopping strategy during training. The RMSE was used as the loss function, and the dropout rate was set to 0.3. The entire model comprised four encoder layers, with the multi-head attention consisting of eight heads. Additionally, excluding the data used for prediction which constituted the test set, 80% of the remaining data were used as the training set while the remaining 20% served as the validation set. All the experiments were conducted on a single RTX4090 GPU to ensure the consistency and reliability of the experimental outputs.

In the prediction of the mesoscale SSTa–LHFa coupling coefficients, we used linear trend values and mean coupling coefficient values as evaluation criteria. Furthermore, via HighResMIP simulations, we compared coupling coefficients of the 10 predicted future winters based on the training ERA5 data with those of the earliest 10 winters in ERA5 under global warming.

### 3.2. Comparison of RMSE

Considering 2008–2017 data as the test set, we used the trained iTransformer model to predict mesoscale SSTa–LHFa coupling coefficients and calculate the linear trend of this interaction for each WBC. Thus, we obtained two sets of models: one based on the use of the original mesoscale SSTa–LHFa coupling coefficients as model inputs for prediction, and another based on the use of coupling coefficients and SST data from the same period as inputs. Given that the variability of mesoscale SSTa–LHFa coupling is mainly dominated by large-scale background SST values, we made reference to existing strategies for improving prediction performance. The RMSE of the mesoscale SSTa–LHFa coupling coefficients for the period 2008–2017, obtained from the two model configurations, are summarized in Table 1. From this table, it is evident that including SST data during the training process resulted in a significant decrease (3.0%) in the RMSE. Hence, subsequent analyses were performed based on training involving the coupling coefficients and SST data.

### 3.3. Predicted Coupling Coefficients and Linear Trend

**Figure 4:**
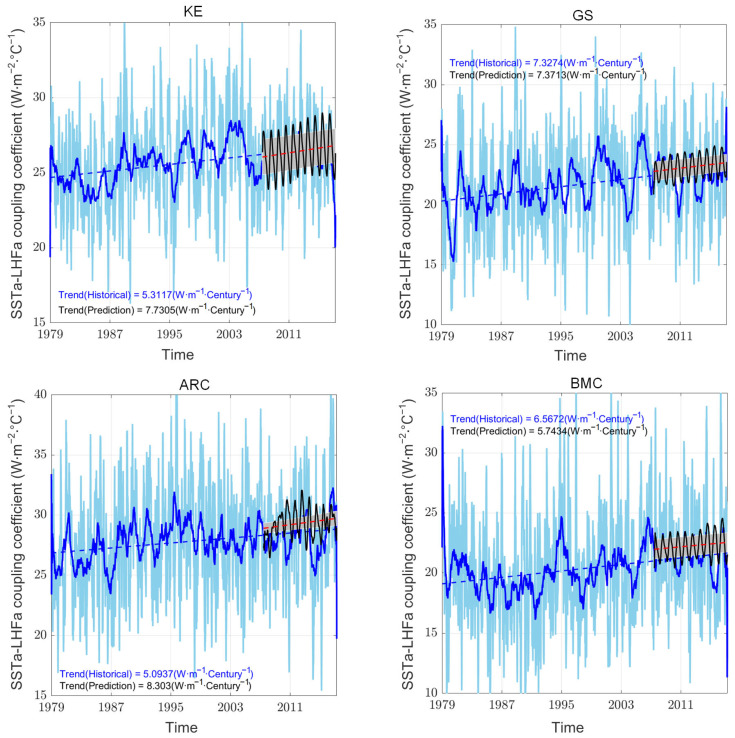
Time series of mesoscale SSTa–LHFa coupling coefficient in WBCs obtained from ERA5 (light blue line) and corresponding linear trend (dark blue dashed line). The tests set with training coupling coefficient and SST simultaneous. The black curve and red dashed line represent the time series of tests coupling coefficient and the linear trend. The dark blue and black curved lines are given 90 days running mean for a better visualization. The linear regression coefficient is significant at 5% level.

**Figure 5:**
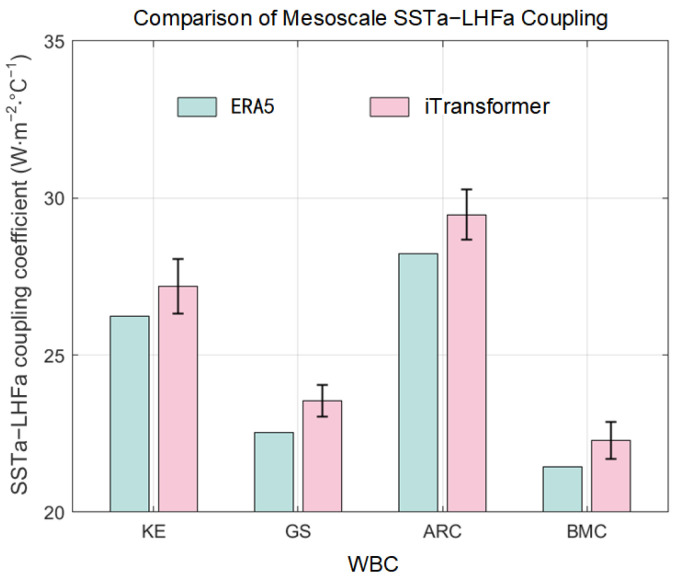
Mean value of mesoscale SSTa–LHFa coupling coefficient in ERA5 and using iTransformer during 2008–2017. Errorbar denotes the standard deviation of test sets.

Based on ERA5 datasets, the mesoscale SSTa–LHFa coupling coefficients exhibited a linear increasing trend from 1979 to 2017 in the four WBCs (Figure 4). Each WBC had 15 ensembles of test sets. Additionally, we calculated the correlation between the model outputs and the corresponding original time series (Table 2). Generally, a good model with strong temporal relationship extraction capabilities and a larger look-back window size should perform well; however, this is not always the case: while increasing the window size can provide more information, it also introduces challenges such as overfitting, increased model complexity, and the potential for noise accumulation, all of which may degrade model performance. It is necessary to select an appropriate look-back window size that corresponds to the characteristics and length of the training time series in the different WBCs. Therefore, based on RMSE values and correlation coefficients, samples that satisfied the following conditions: RMSE below the 40th percentile and correlation coefficient above the 60th percentile and a positive linear trend were retained for subsequent analysis (Table 1 and Table 2). As a result, 4, 4, 4, and 3 of the 15 ensembles for each WBC were selected for KE, GS, ARC, and BMC, respectively. The set means and linear trends (the black solid and red dashed lines, respectively) of the most recent 10 winter coupling coefficients are shown in Figure 4. After filtering based on RMSE values and correlations, the iTransformer model showed comparable linear trends for the mesoscale SSTa–LHFa coupling coefficients in the four WBCs. This observation implied a strong linear trend in the training process. Additionally, the mean value of the predicted coupling coefficients of the latest 10 winters was comparable to those obtained using the original ERA5 dataset (Figure 5).

### 3.4. Future Predictions for 2018–2027

**Figure 6:**
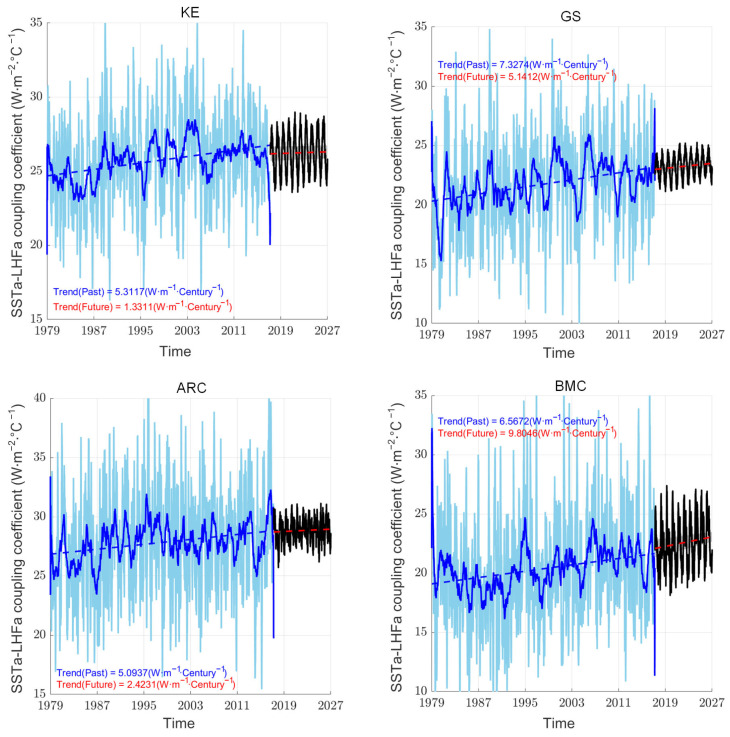
Time series of mesoscale SSTa–LHFa coupling coefficient in WBCs obtained from ERA5 (light blue line) and corresponding linear trend (dark blue dashed line). For the tests sets which training coupling coefficient with adding SST. The black curve and red dashed line represent the time series of tests coupling coefficient and the linear trend training with adding SST for the future 10 winters. The dark blue and black curved lines are given 90 days running mean for a better visualization. The linear regression coefficient is significant at 10% level.

**Figure 7:**
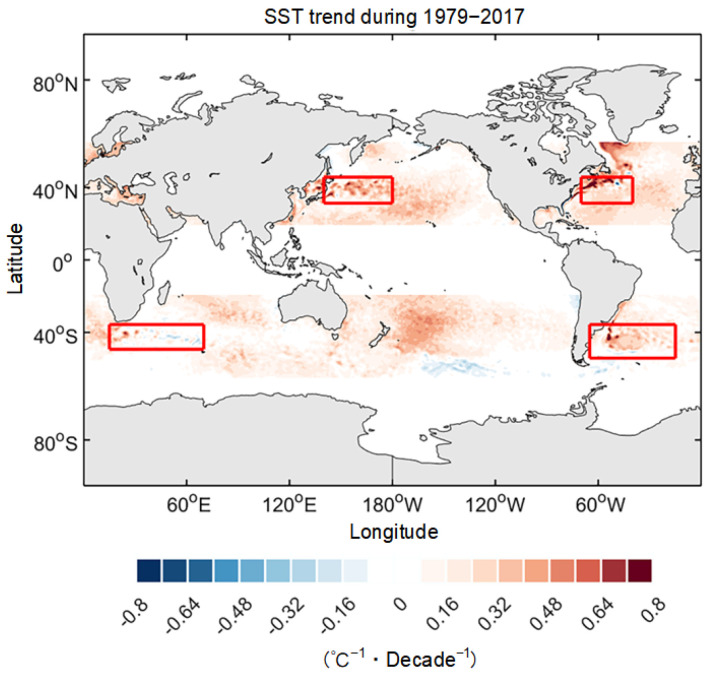
Mid-latitude distribution of the decadal trends of SST as derived from ERA5 during 1979–2017. Shown are the trend of winter season mean and the four WBC regions are outlined by red boxes.

**Figure 8:**
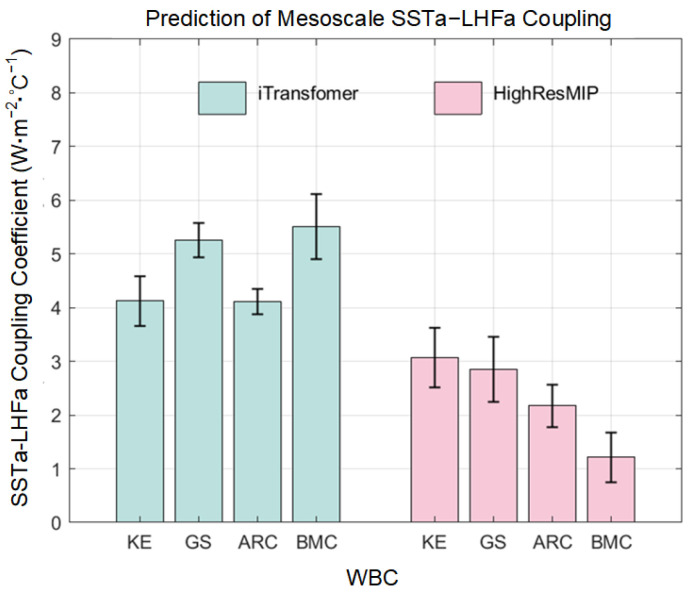
Difference in mesoscale SSTa–LHFa coupling coefficient between 2018–2027 and 1979–1988 based on ERA5 using iTransformer model and the response under global warming based on HighResMIP simulations mean (2041–2050 minus 1950–1959).

Mesoscale SSTa–LHFa coupling is a pivotal modulator and coupler in mid-latitude air–sea interaction systems. Even though we reproduced the variations of mesoscale SSTa–LHFa coupling coefficients for the latest 10 winters, those for the future 10 winters remained unclear. Mesoscale thermal coupling is closely associated with future changes in air–sea interaction. Therefore, we made further predictions for 10 winters after 2017 (2018–2027). The lengths of the selected test sets used to predict mesoscale SSTa–LHFa coupling for the 10 future winters were consistent with those from historical training. Based on training sets consisting of mesoscale SSTa–LHFa coupling coefficients and SST from ERA5 datasets, the prediction showed a significant increasing trend in the KE, GS, ARC, and BMC from 2018 to 2027 (Figure 6). The ensemble’s mean of the mesoscale SSTa–LHFa coupling coefficient time series and its corresponding linear trend over the next 10 winters are shown in Figure 6 (the black solid and red dashed lines, respectively). Additionally, we calculated differences in the mean values of mesoscale SSTa–LHFa coupling coefficients between the 2018–2027 and 1979–1988 periods. We observed that the SSTs of the WBCs increased linearly over the past 39 years under the global warming scenario (Figure 7). Furthermore, we determined the linear trend obtained using the ERA5 dataset via HighResMIP simulations (2041–2050 (RCP) minus 1950–1959 (HIS)). The difference between the 2041–2050 and 1950–1959 periods, in this regard, based on the HighResMIP simulations, represented the response of mesoscale SSTa–LHFa coupling to global warming in scenario RCP8.5 (Figure 8). Additionally, the mean values of the different WBCs were determined using five mesoscale resolving HighResMIP simulations, namely CMCC-CM2-VHR4 (0.25° atmosphere and 0.25° ocean), MPI-ESM1-2-XR (0.5° atmosphere and 0.5° ocean), HadGEM3-GC31-HH (0.5° atmosphere and 0.5° ocean), EC-Earth3P-HR (0.5° atmosphere and 0.25° ocean), and CNRM-CM6-1-HR (0.5° atmosphere and 0.5° ocean).

## 4. Discussion

In this study, we used a newly developed iTransformer model to train mesoscale SSTa–LHFa coupling coefficients derived from high-resolution ERA5 reanalysis datasets. Based on the test set, the model satisfactorily reproduced the mean value and linearly increasing trend in coupling coefficients. Furthermore, including SST in the training process resulted in a significant decrease in the RMSE. In predicting future changes, the model also showed a significant increasing trend in mesoscale SSTa–LHFa coupling coefficients in the WBCs. Additionally, the increasing trend was stronger than those obtained via HighResMIP simulations in a warming climate scenario and the last segment of the data used for training contributed more to the output results. Although the RMSE and correlation coefficients obtained via the HighResMIP simulations were unsatisfactory, their use in data selection was necessary, and, considering the length and number of training sets, as well as the linear enhancement trend of the predicted time series, was necessary as the input lengths of the training sets chosen, which differed across the various WBCs. Even though a large number of training sets were used, the addition of 2008–2017 data to the training resulted in a non-significant linear enhancement trend. Regardless, these data had a non-negligible impact on the final output results for the 2018–2027 period, especially in KE. This is an inevitable issue that is associated with training involving distant to recent data (1979–2017). From a climate prediction perspective, a training set with a shorter input length may not be very suitable for forecasting periods of 10 years or more. In LTSF tasks, the use of short-term data to predict long-term data has already been employed as an application scenario to compare the predictive performance of models, and the iTransformer has shown state-of-the-art predictive performance with respect to real-world forecasting benchmarks [41].

The existence of the aforementioned issues necessitates that more attention be paid to the inherent characteristics of time series data when tailoring training plans. In this study, we primarily focused on the ability to reproduce the linearly increasing trend of time series data. The training method employed, to some extent, demonstrated the ability to estimate future trends based on the linear trend of the data used. We predicted only linear trends, whereas climate variability is characterized by multiple trends. Furthermore, this method can only assume that future changes will continue to increase linearly, as has been observed for historical periods. Therefore, under this assumption, we could only perform a historical analysis, which produced results more reliable than those obtained through HighResMIP simulations under global warming. This finding may be attributed to the retained training results, which exhibited a linearly increasing trend.

Recent studies have revealed future changes in mesoscale thermal coupling from the perspective of physical mechanisms. However, the stable simulation of the response of air–sea systems to changes in thermal coupling coefficients using a high-resolution mesoscale resolving climate model requires a long processing time. Although such stable simulations are achievable through high-resolution simulations, in this study, we successfully reproduced the linear trend of mesoscale SSTa–LHFa coupling coefficients in a short time. First, we constructed and verified the iTransformer model without high-resolution mesoscale-resolving climate model simulation. Second, our results indicated that the constructed iTransformer model can be expanded to perform long-term climate predictions in a shorter time. Additionally, our findings suggest that a larger number of climatic variables can be used as training sets for prediction. For example, in the North Pacific Ocean, it was possible to simultaneously train variations in mesoscale air–sea interaction, storm tracks, North Pacific decadal oscillations, and Aleutian low to the end of visualizing possible future changes in this ocean. While the iTransformer model effectively performed well overall in the four WBCs, its performance in other regions and climate conditions is uncertain. Our future studies will test its adaptability across different regions and climates.

## 5. Conclusions

In this study, we used an iTransformer model to predict mesoscale SSTa–LHFa coupling in WBCs for up to 10 future winters. The model demonstrated satisfactory performance, and incorporating SST data into the training process significantly improved its performance by reducing the RMSE. The correlation between the test sets and the original time series varied with the length of the training sets. Furthermore, the retained test set obtained after filtering based on RMSE and correlation sufficiently reproduced coupling coefficients (mean values and linear trends) based on the ERA5 datasets. Furthermore, making reference to the independent training scheme for the latest 10 winters in the four WBCs, we made predictions for 10 future winters and estimated the response of mesoscale SSTa–LHFa coupling to global warming. Via HighResMIP simulations under global warming scenarios, we observed a consistently intensified mesoscale SSTa–LHFa coupling strength; however, our results showed regional dependence, i.e., the coupling coefficients were stronger in the Northern Hemisphere, an observation that is unsatisfactory. Thus, further studies are required in this regard. Regardless, the iTransformer model showed satisfactory performance in terms of the ability to reproduce the linear trends and mean values of coupling coefficients for the four WBCs and the prediction of mesoscale thermal coupling for 10 future winters. This model provides a new pathway for exploring mesoscale air–sea interaction variations in future climate change predictions. Additionally, the model can be extended to other climate-related predictions based on long-term historical observation datasets in cases where evidence from climate models is lacking.

## Figures and Tables

**Table 1:** RMSE of outputs of mesoscale SSTa–LHFa coupling time series in ERA5 during 2008–2017. For each WBC, the RMSE is presented for two distinct experimental setups: (1) the iTransformer model with inputs of both the SSTa–LHFa coupling coefficient and SST, and (2) the iTransformer model with inputs of the SSTa–LHFa coupling coefficient alone.

WBC	Variable	Look-Back Windows														
		1	2	3	4	5	6	7	8	9	10	11	12	13	14	15
KE	Coupling Coefficient + SST	4.62	4.43	4.42	4.47	4.59	4.68	4.83	4.72	4.62	4.82	4.82	4.92	4.82	4.86	4.77
	Coupling Coefficient	4.77	4.66	4.40	4.53	4.66	4.59	4.67	4.70	4.69	4.71	4.81	4.92	5.10	5.34	5.05
GS	Coupling Coefficient + SST	6.31	6.56	6.52	6.40	6.47	6.49	6.60	6.74	6.61	6.72	6.67	6.77	6.91	6.83	7.05
	Coupling Coefficient	6.40	6.78	6.63	6.51	6.46	6.56	6.70	6.79	6.79	6.81	6.59	6.99	7.06	7.34	7.28
ARC	Coupling Coefficient + SST	7.94	7.46	7.70	7.67	7.48	7.80	7.59	7.72	7.86	7.91	7.97	8.03	8.11	8.15	8.26
	Coupling Coefficient	7.94	7.66	7.80	7.58	7.81	7.94	7.90	7.82	8.17	8.14	8.08	8.27	8.32	8.29	8.33
BMC	Coupling Coefficient + SST	7.35	7.32	7.23	7.21	7.54	7.49	7.54	7.41	7.49	7.48	7.46	7.66	7.86	7.76	7.74
	Coupling Coefficient	7.55	7.63	7.40	7.78	7.86	7.76	7.88	8.38	7.99	8.06	8.23	8.20	8.83	8.29	8.21

**Table 2:** Correlation between iTransformer model outputs and real mesoscale SSTa–LHFa coupling time series in ERA5 during 2008–2017 with training coupling coefficient with adding SST.

WBC	Look-Back Windows														
	1	2	3	4	5	6	7	8	9	10	11	12	13	14	15
KE	0.66	0.63	0.67	0.69	0.67	0.66	0.56	0.63	0.68	0.66	0.63	0.61	0.60	0.57	0.61
GS	0.26	0.23	0.13	0.17	0.14	0.14	0.17	0.18	0.03	0.07	0.08	0.12	0.08	0.11	−0.03
ARC	0.15	0.15	0.24	0.28	0.27	0.17	0.27	0.19	0.22	0.22	0.23	0.16	0.12	0.08	−0.03
BMC	0.43	0.31	0.36	0.28	0.16	0.34	0.29	0.27	0.14	0.19	0.26	0.18	0.15	0.22	0.27

## Data Availability

ERA5 post-processed daily statistics on single levels from 1940 to present dataset can be downloaded from https://doi.org/10.24381/cds.4991cf48 (accessed on 14 October 2024). The HighResMIP data can be downloaded from https://pcmdi.llnl.gov/CMIP6/ (accessed on 22 November 2016).
